# KLIFS: an overhaul after the first 5 years of supporting kinase research

**DOI:** 10.1093/nar/gkaa895

**Published:** 2020-10-21

**Authors:** Georgi K Kanev, Chris de Graaf, Bart A Westerman, Iwan J P de Esch, Albert J Kooistra

**Affiliations:** Division of Medicinal Chemistry, Amsterdam Institute for Molecules, Medicines and Systems (AIMMS), Vrije Universiteit Amsterdam, De Boelelaan 1108, 1081 HZ Amsterdam, The Netherlands; Department of Neurosurgery, Amsterdam University Medical Centers, Cancer Center Amsterdam, Brain Tumor Center Amsterdam, De Boelelaan 1117, 1081 HV Amsterdam, The Netherlands; Sosei Heptares, Steinmetz Building, Granta Park, Great Abington, Cambridge, CB21 6DG, UK; Department of Neurosurgery, Amsterdam University Medical Centers, Cancer Center Amsterdam, Brain Tumor Center Amsterdam, De Boelelaan 1117, 1081 HV Amsterdam, The Netherlands; Division of Medicinal Chemistry, Amsterdam Institute for Molecules, Medicines and Systems (AIMMS), Vrije Universiteit Amsterdam, De Boelelaan 1108, 1081 HZ Amsterdam, The Netherlands; Department of Drug Design and Pharmacology, University of Copenhagen, Universitetsparken 2, 2100 Copenhagen, Denmark

## Abstract

Kinases are a prime target of drug development efforts with >60 drug approvals in the past two decades. Due to the research into this protein family, a wealth of data has been accumulated that keeps on growing. KLIFS—Kinase–Ligand Interaction Fingerprints and Structures—is a structural database focusing on how kinase inhibitors interact with their targets. The aim of KLIFS is to support (structure-based) kinase research through the systematic collection, annotation, and processing of kinase structures. Now, 5 years after releasing the initial KLIFS website, the database has undergone a complete overhaul with a new website, new logo, and new functionalities. In this article, we start by looking back at how KLIFS has been used by the research community, followed by a description of the renewed KLIFS, and conclude with showcasing the functionalities of KLIFS. Major changes include the integration of approved drugs and inhibitors in clinical trials, extension of the coverage to atypical kinases, and a RESTful API for programmatic access. KLIFS is available at the new domain https://klifs.net.

## INTRODUCTION

**Figure 1.**
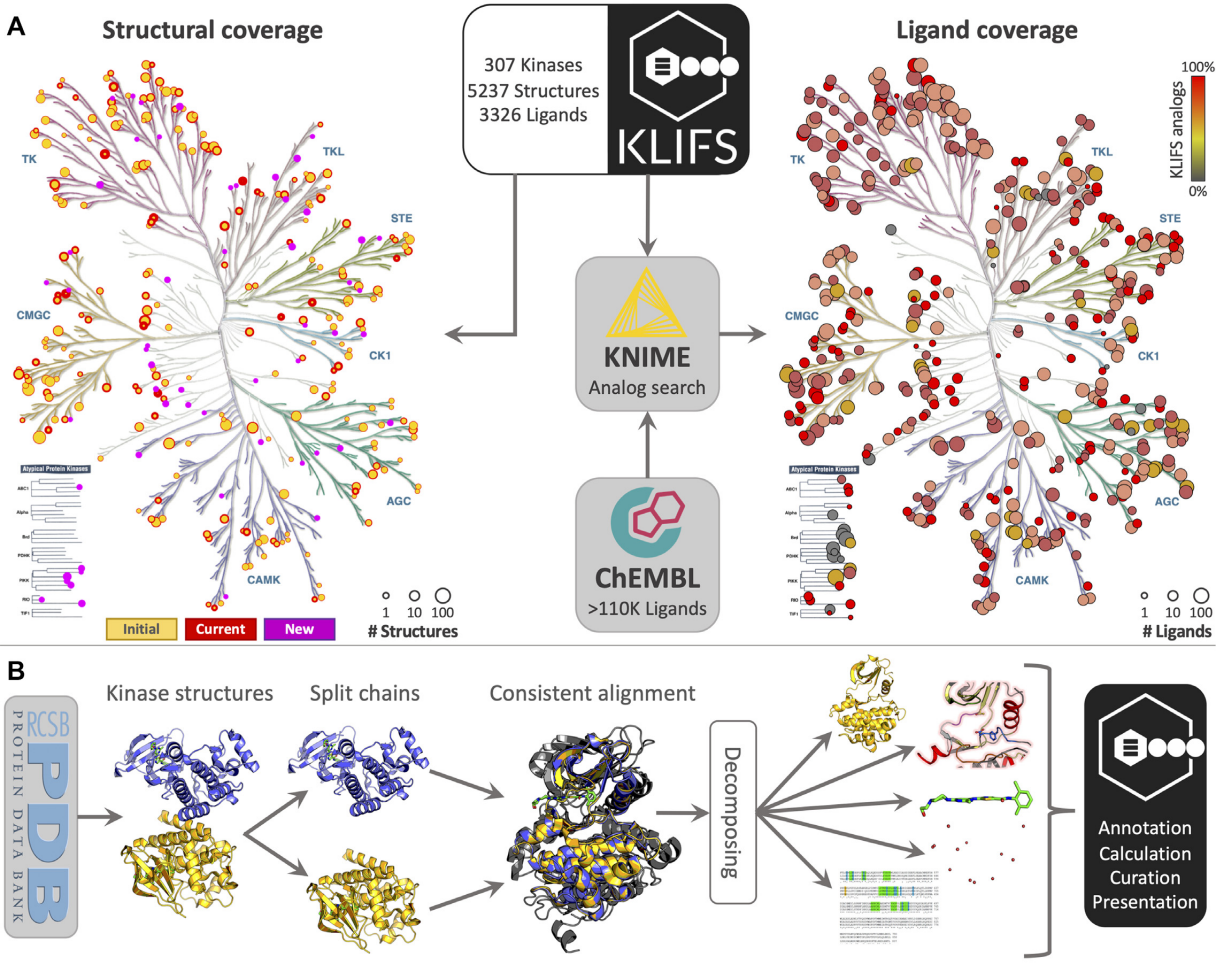
An overview of the pipeline and contents of KLIFS. (**A**) The left kinome shows the current structural coverage of KLIFS for each kinase. The size of the circles indicates the number of structures for each kinase with in purple the newly covered kinases, in red the current status of previously covered kinases with in yellow on top the status at the time of the previous publication (4). The right kinome shows the ligand coverage based on ChEMBL and is colored according to the % of ligands that are analogs of the inhibitors present in KLIFS. Of note, the circles represent the number of structures and ligands on a logarithmic scale. The kinome visualizations were created using KinMap (44). (**B**) A schematic depiction of the pipeline of how the structures are processed before their annotation, calculation, curation and presentation in KLIFS.

The pivotal role of kinases as modulators of cell signaling has made them a primary target for drug discovery efforts. In the past two decades, this effort has led to the FDA approval of 64 small molecule kinase inhibitors of which >80% in the last decade (1,2). Moreover, the undertakings of the research community have resulted in a wealth of data in the public domain with thousands of kinase structures, hundreds of thousands of kinase inhibitors, millions of inhibitor-kinase bioactivity data points, and much more (3). Therefore, to support structure-based kinase research amidst this wealth of data, the KLIFS—Kinase–Ligand Interaction Fingerprints and Structures—database was developed (4,5). KLIFS focuses on how small molecule kinase inhibitors interact with the catalytic kinase domain. To this end, KLIFS collects all kinase structures and aligns, decomposes, annotates, curates, and enriches them with related kinase information (Figure 1B).

Since the introduction of the KLIFS website 5 years ago (4), KLIFS has grown both in terms of content as well as user base. The user base has steadily grown with currently >400 unique visitors and over ten thousand pageviews on a monthly basis. With the weekly updates, the content of KLIFS keeps growing. The amount of kinase structures has almost doubled: KLIFS now (1 August 2020) contains over 5200 annotated kinase structures comprising 307 unique kinases (including atypical kinases (6)) and >3300 unique inhibitors (Figure 1A). While a lot of structural data remains undisclosed by pharma and biotech companies, the inhibitors in KLIFS appear to be good representatives of all known kinase inhibitors as became clear from a simple 2D chemical similarity analysis against kinase inhibitors in the ChEMBL database. The similarity analysis revealed that while the number of ligands is ∼2% of the known kinase inhibitors in ChEMBL, they are analogs (defined as an ECFP-4 similarity ≥ 0.4) of nearly 60% of all kinase inhibitors in ChEMBL (Figure 1A). This shows that even without the identical ligand being present in KLIFS, related crystallized ligands can provide valuable information on how that ligand binds and interacts with the catalytic cleft (7). Besides, the application of more advanced *in-silico* similarity methods would even further increase the coverage.

Ever since its introduction, the community has used KLIFS for a wide variety of applications, far beyond the initial applicability domain we envisioned upon creating the database. Here, we highlight a small selection of the applications from the community. Hao *et al.* (8) utilized KLIFS to support the rational design of selective PAK4 inhibitors. Researchers from Janssen R&D describe in their work (9) a set of their protocols for kinase drug discovery, including a workflow using KLIFS to rationally design a combinatorial database of potential kinase inhibitors based on, amongst others, inhibitor type and pharmacophore properties. Sorrell et al. (10) rationalized the conservation of binding modes in inhibitors binding to members from both the JAK and NAK family using KLIFS. Klaeger *et al.* (11) extensively screened nearly all clinical kinase inhibitors against hundreds of kinases and used KLIFS to classify the binding mode of these inhibitors. For 137 inhibitors they found binding mode information in KLIFS based on which they could confirm that kinase inhibitors of type II (DFG-out) are not by default more selective than the DFG-in Type I inhibitors. Georgi *et al.* (12) experimentally evaluated the binding kinetics of 270 inhibitors against 40 kinases and used KLIFS to identify possible determinants of a particular binding kinetics profile. Inhibitors that bound in a DFG-out conformation generally had a longer residence time compared to DFG-in binders. Moreover, they also evaluated links between kinase-ligand interactions and the residence time and identified interactions with residues at specific locations that affected the on and off rates. Also focused on binding kinetics, Uitdehaag *et al.* (13) found that the most potent TTK inhibitors with an increased residence time induced a shift in the G-rich loop by interacting with the conserved lysine (K^III.17^), and named this the ‘lysine trap’. Using KLIFS, they identified other structures in which the inhibitors portrayed this lysine trap mechanism. Dr Robert Roskoski Jr has utilized KLIFS in multiple in-depth reviews, e.g. (2,14,15), to describe the binding pocket of kinases, to identify crucial residues in the binding site and to describe the hydrophobic spines. In addition, in his reviews, he extensively uses the KLIFS residue nomenclature and kinase-ligand interactions to describe how clinically relevant kinase inhibitors interact with their target.

The KLIFS residue nomenclature, structures (https://doi.org/10.7490/f1000research.1117262.1), structural annotations, and kinase-ligands interactions have also been used by several as input for machine learning models related to kinases and kinase inhibitors or for the creation of curated datasets for machine learning. Both Sorgenfrei *et al.* (16) and Jaeger et al. (17) used the KLIFS binding site sequence alignment to generate protein descriptors in order to create predictive models for *in silico* kinase profiling of small molecules. Miljković *et al.* (18) used the KLIFS binding mode annotations to create an annotated reference dataset in order to train classifiers to predict the type of inhibitor, based solely on molecular fingerprints. Rodríguez-Pérez *et al.* (19) extended on that study and evaluated both the KLIFS interactions as well as a molecular fingerprint and a combination of the two for the classification of binding modes.

In this article, we provide an overview of all updates of KLIFS, introduce the new website and the OpenAPI web services, and showcase a few of the newest features.

## METHODS

### Updating the bioactivity data

From the ChEMBL database (20), a major open resource with manually curated bioactivities for small molecules, KLIFS collects bioactivity data (21) for all ligands that are present in the kinase structures processed by KLIFS. The bioactivity data is subsequently filtered for all *K*_d_, *K*_i_, EC_50_, IC_50_ entries with a defined pChembl value, a confidence score ≥8, and where the ‘standard_relation’ of the entries is set to ‘ = ’. See the ChEMBL documentation for more information on these terms. Subsequently, the remaining bioactivities are filtered for all protein targets that are present in KLIFS (based on their UniProt accession codes) or where the target family classification is defined as ‘Kinase’ within ChEMBL. With the weekly updates of KLIFS, the bioactivities are collected for all new ligands. Upon release of a new ChEMBL version, all existing bioactivities are purged and recollected from the latest version as soon as possible.

### Extending to atypical protein kinases

In order to expand KLIFS with atypical protein kinases that have a catalytic domain that is structurally comparable to eukaryotic protein kinases (these atypical kinases are also known as protein kinase-like kinases (6)), the creation of a manually curated reference sequence and structural alignment was crucial. Initially, a manual structural alignment was performed between the existing eukaryotic kinase structural alignment (4) and a set of representative atypical kinase structures (PDB-codes 4HNE, 4JSN, 4KZ0, 4OTP, 4OVU, 4UWH, 4WTV, 5C46, 5DXU, 5I35). Iterative refinement of the sequence alignment based on the structural alignment resulted in the final structural reference. Finally, sequences for all atypical kinases were aligned to the structure-based sequence alignment generating the reference sequence alignment. Since then, the PDB is also weekly screened for new atypical kinase structures, which are now also automatically added to KLIFS.

### Updating nomenclature and linking external databases

Based on the UniProt (22) accession codes annotated in KLIFS, the UniProt is checked for the latest updates. Subsequently, the updated accession codes are used to query the HGNC: HUGO Gene Nomenclature Committee resource (23), the MGD: Mouse Genome Database (24), and the IUPHAR Guide to Pharmacology (25) in order to obtain the latest changes in nomenclature and external IDs. The MGD was newly added since the previous publication in order to provide curated gene names for the mouse kinases similar to the HGNC gene names.

## KLIFS: NEW LOOK, NEW LOGO, NEW FEATURES

For this lustrum, the KLIFS website underwent a complete overhaul. The new and clean interface will hopefully provide for a better user experience, and it is now much more responsive to the type of device and its screen size. On modern mobile devices, the website is automatically adjusted to the smaller device dimensions and can handle touch events. In addition to the website layout, also the logo of KLIFS has been renewed (Figure 1) and the domain has moved to https://klifs.net. Of note, the previous domain will remain active for two more years and is forwarded automatically to the new domain. In the next segment, we will provide an overview of the new features and updated content of KLIFS.

### Atypical kinases

Besides structures of eukaryotic protein kinases, KLIFS now also covers all atypical protein kinases (see methods) that share the archetypical eukaryotic protein kinase fold (also known as protein kinase-like kinases). An in-depth analysis of the similarities and dissimilarities of eukaryotic and atypical protein kinases using KLIFS is available here (6). The addition of this kinase class to KLIFS allows for the direct comparison of kinases and inhibitors across these kinase classes. This opens up new opportunities for the design of inhibitors that target both atypical and eukaryotic protein kinases, a combination that is often found to provide synergistic anticancer effects (26,27).

### Quick search

The menu system at the top of the website contains a quick search option. Where this search option could previously solely handle searches for a single PDB-code, the new search option provides results for kinases, kinase names, partial and full PDB-codes, and kinase inhibitor codes and names. Depending on the query, one or more search results are dynamically presented to the user, which allows one to quickly identify the kinase structures of interest.

### Search

The search page allows the user to perform search queries varying from very simple to highly complex searches through the combination of the multiple search options. These search options cover the kinase classification (name, family, group, and species), the inhibitor (physicochemical properties, chemical similarity, and the presence of a substructure), the interactions between the kinase and the ligand (interaction fingerprint), and the structure of the kinase (structural properties, kinase conformation, pocket sequence, water molecules). To assist in searching for inhibitors with specific physicochemical properties, we have added buttons that automatically adjust the search options to predefined sets of rules for physicochemical properties (Rule of 5, Rule of 3, Lead-like and Fragment-like properties). The structural properties have been extended with the option to search for structures that were released in a specific period.

### Search results

After the user has performed a search, all entries in KLIFS that match the search filter(s) are presented in a sortable results table. The user can view the chemical structure of the inhibitor by hovering over the name of the ligand, and the coverage of the kinase superfamily can be plotted onto the kinome highlighting the kinases for which structures were identified. The results table is now a dynamic table that is split across multiple pages and is fully searchable and sortable. All results can be exported to CSV or Excel and all structures can also be downloaded as a package. The ‘Download Structures’ option has been extended with a PDB download, in addition to the existing MOL2 and MOE (Chemical Computing Group Inc.) download options.

### Structure details

Each structure has a detailed overview page in which all the information about this single structure is presented. At the top, information about the structure of interest and the crystallized kinase is provided together with a download icon that gives the option to download a PDB or MOL2 file of the structure as processed and aligned by KLIFS. An advantage of this is that when multiple downloaded structures are simultaneously opened in a molecular viewer, all structures are aligned based on the catalytic cleft and can be easily compared. Another new addition is the ability to download a PyMOL session (PyMOL Molecular Graphics System – Schrödinger, LLC), which contains the kinase and ligand in the same view as provided in KLIFS together with the KLIFS coloring for the binding pocket. Underneath the structural information, the interactive 3D NGL (28) viewer shows the kinase structure, but can also be toggled to show pre-generated 2D PyMol images of the front and top of the kinase with surface cut-throughs focusing on the binding pocket. The NGL viewer can, if desired, be viewed in full screen and shows both the structure residue numbering together with the KLIFS residue numbering when hovering over a residue. Residues can be shown or hidden by clicking on the position in the cartoon, which will toggle a stick view of the selected residue. This viewer allows for the quick exploration of the kinase structure and, together with the KLIFS coloring and numbering, can assist in the identification of key residues.

Orthosteric and allosteric ligands are now shown separately, and both have an overview of all known kinase bioactivities in ChEMBL when available (see methods). In addition, a button under the image of the structure allows for a quick search for analogs of that ligand in KLIFS by executing a similarity search (Morgan fingerprint with a minimum Tanimoto similarity score of 0.4). A new schematic view of the catalytic cleft is now provided for the orthosteric ligand and highlights the residues with which the ligand interacts and are colored based on the type of interactions. This schematic view can also be downloaded as an image and the coloring and numbering can be customized.

### Browse

This page gives the user the opportunity to go through all entries in KLIFS without any form of filtering or searching. Similar to the renewed search results page, this page now also shows a dynamic table that is easy to filter, search and sort.

### Drugs

This new page provides an overview of drugs and clinical candidates that target kinases. This data is obtained from the PKIDB (1,29), a resource dedicated to providing an up-to-date and manually curated overview of approved kinase inhibitors and clinical candidates. The list of compounds acquired from the PKIDB is screened for available data within ChEMBL using the readily provided ChEMBL IDs and the available bioactivity information is linked to each compound in KLIFS. Based on the molecular structure as obtained from the PKIDB, the user can quickly identify structures in complex with a specific inhibitor or find structures with (close) analogs of the inhibitor. The analog search is performed using the Morgan fingerprint, as implemented in the RDkit cartridge (RD-Kit: Open-source chemoinformatics; http://www.rdkit.org), for a similarity screening (a minimum Tanimoto similarity score of 0.4) against all ligands in the KLIFS database.

### RESTful web services

Initially, the data from KLIFS was solely available via download options using the web interface. However, to enable also programmatic access to the KLIFS data for more data-intensive and customized setups we have developed RESTful web services (reference is available at https://klifs.net/swagger/). The web services have been implemented according to the OpenAPI specifications (https://github.com/OAI/OpenAPI-Specification) and are therefore compatible with, amongst others, the Swagger Tools (https://swagger.io). Based on the specification (available at https://klifs.net/swagger/swagger.json), programmers can automatically generate client SDKs to communicate with KLIFS in their own programs. The open-source tool Swagger CodeGen, for example, can be used to automatically generate a client SDK based on the KLIFS specifications for more than 50 different frameworks in 40 different programming languages. Moreover, the KLIFS nodes (30,31) for KNIME (32) also make use of the web services to obtain their data. KNIME (32) is an easy-to-use open-source data analytics platform that amongst others enables cheminformatics analyses. These nodes make it possible for any user, also non-programmers, to create advanced structural bio- and cheminformatics pipelines (33). Examples of such pipelines include structure-based kinase bioactivity data mapping, structure-based identification of scaffold replacements for kinase inhibitor optimization, and structure-based pharmacophore comparison for ligand repurposing (31).

## KLIFS: SUPPORTING STRUCTURE-BASED KINASE RESEARCH

### Powerful web-based search queries

With the new website, the search function is now more powerful than before. By mixing multiple search options, the user can perform complex search queries with the click of a few buttons. Here we showcase three search queries, ranging from simple to more complex, that are linked to recent advances in kinase research.

#### Understanding the binding mode of a drug without a structure

**Figure 2.**
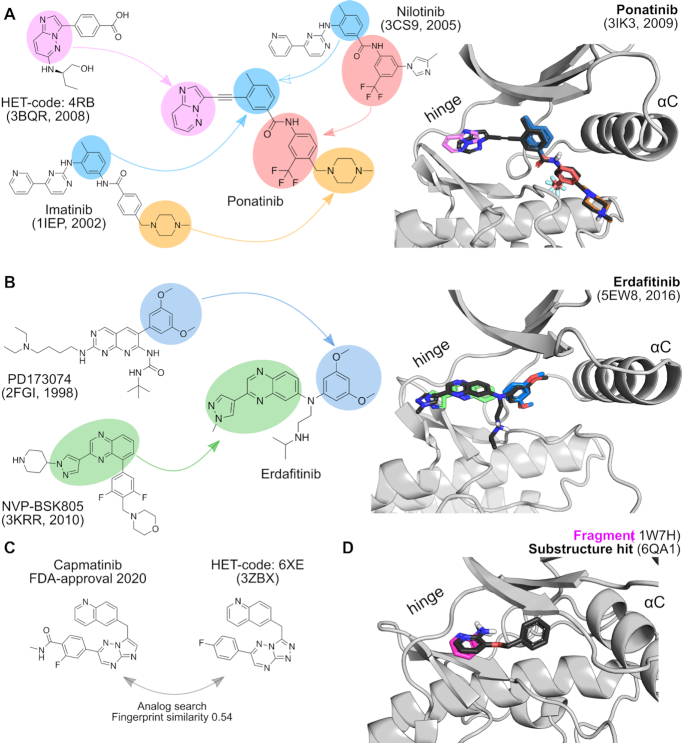
(**A**) Partial reconstruction of Ponatinib using fragments from structures released before 2009. Fragments from Imatinib, Nilotinib and HET-code 4RB are shown together on top of the binding mode of ponatinib within the kinase binding site. (**B**) Partial reconstruction of Erdafitinib based on fragments from structures released before 2016. Fragments from PD173074 and NVP-BSK805 are shown on top of the binding mode of Erdafinitib within the kinase binding site. (**C**) An analog search for the recently approved inhibitor Capmatinib identified HET-code 6XE as the most similar inhibitor in KLIFS with a fingerprint similarity of 0.54 (Tanimoto score using the Morgan fingerprint). (**D**) An overlay of a structure with a hit from an ultra-small fragment screen with a substructure search hit within KLIFS. This overlay shows a conservation of the binding pose of the hinge-binding fragment in 6QA1 with the larger ligand with this fragment as substructure as observed in 1W7H.

The new drugs page provides an overview of drugs and clinical candidates as obtained from the PKIDB (1), thereby enabling the use of this information from within KLIFS. Take, for example, the recently approved kinase inhibitor capmatinib (FDA-approval 6 May 2020). From the drugs page, it is directly clear that there are currently no kinase structures in complex with capmatinib available, but there is bioactivity data available for its primary target (visible when clicking on the ‘Show bioactivity data’ button). By clicking on the ‘Find structures with analogs’, KLIFS uses the chemical structure of capmatinib to perform a similarity screen against all ligands in KLIFS. This query returned a total of three hits, of which the most similar hit is PDB-code 3ZBX; a structure of MET (the primary target of capmatinib) with a close analog. Hovering over the ligand name displays the chemical structure of the drug and the hit side-by-side and shows that these are indeed very close analogs (Figure 2C). The binding mode of this structure will therefore provide a good template to understand the binding and interactions of capmatinib with MET.

#### The molecular determinants of selectivity

Recently, a paper was published by Gerstenberger et al. on the development of the selective TYK2 inhibitor PF-06826647 (34). In their paper, the authors describe having identified a determinant for the lower affinity of JAK inhibitors, such as tofacitinib, baricitinib and ruxolitinib, for TYK2 due to their pyrrolopyrimidine hinge-binding moiety. They noticed that I960^b.l.36^, which is a valine for the other JAK kinases, changes conformation owing to the pyrrolopyrimidine group. They, therefore, aimed to design new inhibitors with improved affinity for TYK2 by utilizing a non-clashing moiety and ended up using a pyrazolopyrazine, which indeed resolved this clash and showed an improved affinity for TYK2 (PDB-code: 6X8G). With the renewed search page in KLIFS, users can perform powerful search queries via the web interface. In this case, we performed a search for TYK2 structures and drew the pyrrolopyrimidine moiety for a combined substructure search. This search resulted in one hit, namely that of tofacitinib bound to TYK2 (PDB-code: 3LXN), which clearly showed the rotamer change of I960^b.l.36^. We wanted to know if this observation holds true for kinases outside of the JAK family and therefore performed a second search. In this second search, we again specified the pyrrolopyrimidine as a substructure and limited the search to kinases with an isoleucine at b.l.36 and a methionine as a gatekeeper (gk.25) similar to the JAK kinases. This search returned five hits, excluding the TYK2 hit, with structures for the kinases TTK, MAPK8, and CHEK1. In all these structures, the specific rotamer conformation for I^b.l.36^ was observed due to the pyrrolopyrimidine. Therefore, this optimization strategy might not just be limited to TYK2 and can potentially also be applied to these kinases.

#### Postulating the binding mode of substructures

In recent work from O’Reilly et al. the authors published the results of crystallographic screens against MAPK1 (Erk2) using very small fragments, with compounds of just 5–7 heavy atoms (35). The binding modes of the fragments provide information for the rational design or optimization of MAPK1 inhibitors. With the substructure search option, we can quickly investigate if the hit fragments have also been observed as a substructure in larger ligands. The pyridine-2-amine fragment, for example, was found to bind to seven places in MAPK1 (PDB-code: 6QA1). Five spots were outside of the catalytic cleft, one spot next to the hinge and the last spot near the conserved lysine K54^III.17^. A search for this substructure in members of the MAPK family resulted in 30 unique hits. The oldest of the hits, namely PDB-code 1W7H, is a MAPK14 (p38a) in complex with a larger fragment ligand, 3-(benzyloxy)pyridin-2-amine, for which the pyridine-2-amine moiety shows a close overlay with the hinge-binding fragment in MAPK1 (Figure 2D).

### Scaling up structure-based kinase research with the API

With the RESTFUL API allowing programmatic access to all data in KLIFS, more data-intensive and larger analyses are now supported. In this segment we will showcase two examples, to give a flavor of the kind of studies that are now possible with KLIFS.

#### Fragmenting ligands to rationally construct new inhibitors with binding mode information

As KLIFS aligns all kinase structures, the binding mode of a ligand in one structure can immediately be compared to the binding mode of another ligand. Using the API, we acquired all ligands from KLIFS and fragmented the ligands while conserving their 3D coordinates using eMolFrag (36). This resulted in a large library of fragments that were all still aligned within their respective (sub)pockets within the KLIFS-defined binding site of the catalytic kinase domain. By exploiting the information of where the fragments are located and where they were fragmented, i.e. at what bonds, new potential kinase inhibitors can be rationally designed by fusing the fragments back together (9). In order to see if the binding mode information from the fragments was conserved, we tried to match the fragments to the binding mode of two FDA-approved drugs, while only using fragments derived from structures that were published before the binding mode of the drug was known. The first example is Ponatinib in complex with its primary target ABL1, which was published in 2009 (37) (PDB-code: 3IK3). After fragmenting Ponatinib, the fragments were compared to the fragment library based on structures published before 2009. Fragment hits were based on the structure of Imatinib in complex with ABL1 (2001, PDB-code: 1IEP), Nilotinib also in complex with ABL1 (2008, PDB-code: 3CS9), and 4RB in complex with DAPK3 (2008, PDB-code: 3BQR). Overlaying the individual fragments from these structures with the binding mode of Ponatinib shows how closely they match showing only little deviation (Figure 2A). The second example is of Erdafitinib bound also to its primary target, namely FGFR1 (38) (2016, PDB-code: 5EW8). The pyrazole-quinoxaline moiety binding to the hinge region was also found in a JAK2 structure with the inhibitor NVP-BSK805 bound (2010, PDB-code: 3KRR). For the dimethoxybenzene fragment protruding into the gate area, a match was found for PD173074 in complex with FGFR1 (1999, PDB-code: 2FGI). The individual fragments again closely overlay with the binding mode of Erdafitinib (Figure 2B). These results indicate that KLIFS can be used in fragment-based drug discovery (FBDD), not only to guide fragment-hit growing but perhaps also by applying a ligand deconstruction approach. It has been noted in a recent perspective that reviews successful Fragment-to-Lead programs, that the use of literature data is increasingly used as starting points for drug discovery programs (39) and KLIFS can offer ample opportunities for FBDD approaches in the kinase field. While preparing this manuscript, an in-depth study on the fragmentation of kinase inhibitors using KLIFS was prepublished by Sydow *et al.* (40). The authors created a fragment library (KinFragLib), analyzed the physicochemical properties of the fragments in each of the (sub)pockets, and used the fragments to create a combinatorial library comprising >6 million potential kinase inhibitors.

#### Finding crystallized analogs for a large dataset of inhibitors

The web services also power the KLIFS KNIME nodes (30,31) thereby enabling any user, also non-programmers, to utilize the data behind the KLIFS API to enrich data analyses and cheminformatics workflows. The workflow that was created for this example retrieves a list of kinase inhibitors from ChEMBL and tries to find analogs for each of these inhibitors in KLIFS and to provide a list of PDBs of all structures that have this inhibitor co-crystallized. The workflow started out with retrieving all KDR (VEGFR2) inhibitors with a pIC50 value ≥5 from ChEMBL, utilizing a part of a workflow from TeachOpenCADD (41)). A set of 4547 unique KDR inhibitors was obtained and MACCS and ECFP-4 fingerprints were created and used to screen against all KLIFS ligands. Only the best matching ligand in KLIFS with a minimum Tanimoto similarity for MACCS ≥0.8 and ECFP-4 ≥0.4 was kept for each KDR Inhibitor. Subsequently, all matches were linked to the list of PDB-codes in which the KLIFS ligand is found. A total of 3973 KDR inhibitors were matched with a (close) analog in the KLIFS database, covering 87% of the original dataset. These matches provide relevant information for, for example, annotating the probable binding mode of the KDR inhibitors, identification of good reference structures to start modeling, deducing crucial interactions, and possibly the identification of off-targets for the ligands. Supplementary Figure S1 shows the final workflow, which can be downloaded in full from GitHub (https://github.com/3D-e-Chem/workflows).

## CONCLUSIONS AND OUTLOOK

KLIFS has been brought to the next level with a new website, new features, a RESTful API, and the expanding content. However, we also have to note the invaluable feedback from the KLIFS community, which plays an important role in development and curation of KLIFS. In the years to come, KLIFS will be further enhanced through website updates (e.g. better color schemes that also accommodate the colorblind (42)), extending the structural annotation (e.g. with hydrophobic spines (43)), improving the data curation (e.g. ligand tautomers), and the development of new tools. Moreover, with every newly released kinase structure, the coverage and utility of KLIFS will keep increasing.

## DATA AVAILABILITY

The KLIFS database is available at https://klifs.net and all underlying data is also fully accessible via the OpenAPI-compliant RESTful API (reference is available at https://klifs.net/swagger/). The KLIFS KNIME nodes are available from the ‘KNIME Community Extensions’ update site within KNIME, and the source code can be found at https://github.com/3D-e-Chem/knime-klifs (30).

## Supplementary Material

gkaa895_Supplemental_File
