# Supplementary material for: KLIFS: an overhaul after the first 5 years of supporting kinase research
Source: Nucleic Acids Res. 2020 Oct 21;49(D1):D562–9. doi: 10.1093/nar/gkaa895 (PMC7778968; doi:10.1093/nar/gkaa895)
Supplement: gkaa895_Supplemental_File [file gkaa895_supplemental_file.pdf]

Supplementary information

for

KLIFS: an overhaul after the first 5 years of supporting kinase research

Georgi K. Kanev[1,2], Chris de Graaf[3], Bart A. Westerman[2], Iwan J.P. de Esch[1], Albert J. Kooistra[4,*]

[1] Division of Medicinal Chemistry, Amsterdam Institute for Molecules, Medicines and Systems (AIMMS), Vrije Universiteit Amsterdam, De Boelelaan 1108, 1081 HZ Amsterdam, The Netherlands
[2] Department of Neurosurgery, Amsterdam University Medical Centers, Cancer Center Amsterdam, Brain Tumor Center Amsterdam, De Boelelaan 1117, 1081 HV Amsterdam, The Netherlands
[3] Sosei Heptares, Steinmetz Building, Granta Park, Great Abington, Cambridge, CB21 6DG, UK
[4] Department of Drug Design and Pharmacology, University of Copenhagen, Universitetsparken 2, 2100 Copenhagen, Denmark

* To whom correspondence should be addressed. Tel: +45 35328305 Email: albert.kooistra@sund.ku.dk

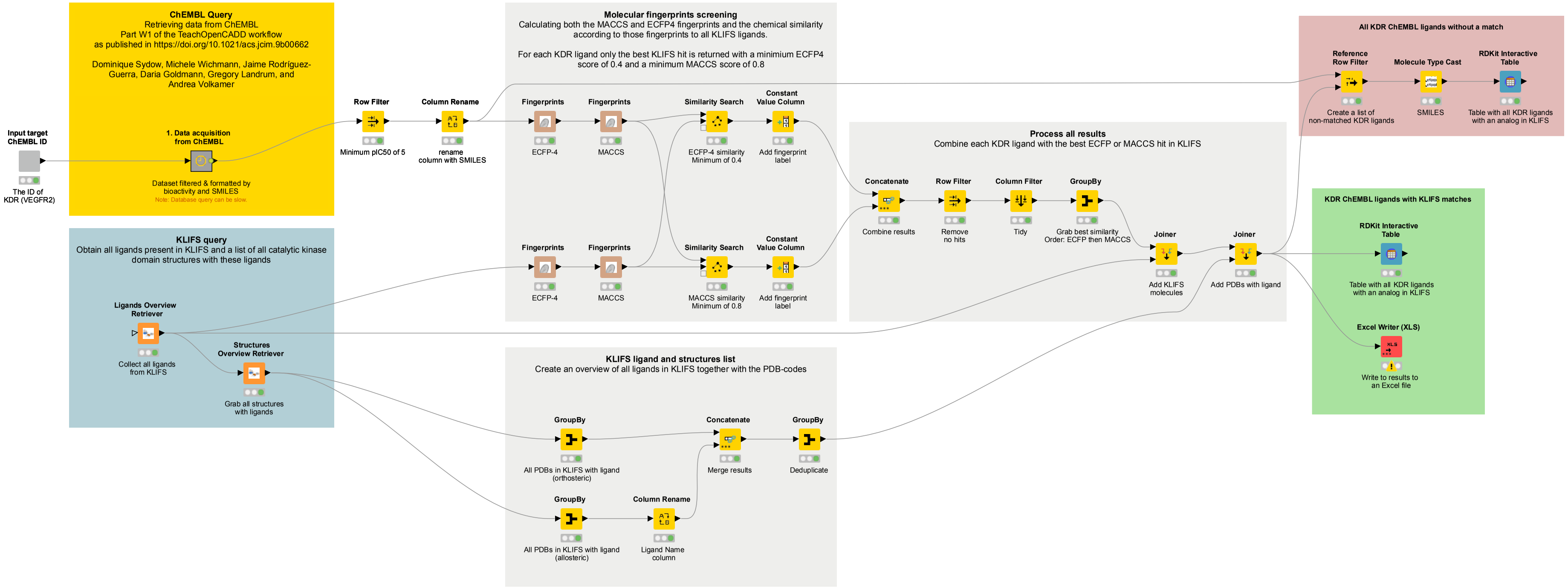

Figure S1. A KNIME workflow utilizing the KLIFS platform to identify analogs within KLIFS for every compound in a compound library in KLIFS. The workflow starts with collecting all KDR (VEGFR2) inhibitors with a pIC50 value ≥ 5 from ChEMBL, followed by a fingerprint similarity analysis (both MACCS and ECFP-4) against all KLIFS ligands as collected by the KLIFS KNIME nodes. All compounds having a (close) analog in KLIFS, defined by a Tanimoto similarity of 0.4 for ECP-4 and 0.8 for MACCS, are linked to the best hit in KLIFS together with a list of PDB-codes in which the ligand is present. The remaining compounds for which no (close) analog was identified are shown in an interactive table. The KNIME workflow can be downloaded at https://github.com/3D-e-Chem/workflows
